# Five-Year Outcomes of Bioresorbable Stent Therapy for Coronary Heart Disease: A Systematic Review and Meta-Analysis of Randomized Controlled Trials

**DOI:** 10.31083/j.rcm2507238

**Published:** 2024-06-28

**Authors:** Fei-fei Yang, Hui Song, Wei-bin Qin, Wei-zhi Tang, Ling-jun Zhan, Li-wen Zhang, Gui-xin He

**Affiliations:** ^1^Graduate School, Guangxi University of Chinese Medicine, 530000 Nanning, Guangxi, China; ^2^The Second Ward of the Department of Cardiovascular Medicine, The First Affiliated Hospital of Guangxi University of Chinese Medicine, 530000 Nanning, Guangxi, China

**Keywords:** bioresorbable stent, coronary heart disease, five-year outcomes, meta-analysis, systematic review

## Abstract

**Background:**

The efficacy of bioresorbable vascular scaffolds (BVS) compared to metallic stents for the treatment of coronary heart disease remains controversial. The analysis of clinical outcomes at five years following the initial treatment has yet to be reviewed. This study sought to assess the five-year outcomes in randomized controlled trials of BVS in the treatment of coronary heart disease using a systematic review and meta-analysis.

**Methods:**

A systematic database search was conducted from their inception to June 30th, 2023 using various Medical Subject Headings (MeSH) terms including: “Coronary Disease”, “Bioresorbable stent”, “Randomized controlled trials”.

**Results:**

After a rigorous selection process, a total of five high-quality articles were finally included in this study. Each trial demonstrated a low risk of bias. After 5 years, bioresorbable stents showed outcomes similar to conventional metal stents in terms of cardiac mortality. However, they were inferior in terms of lesion revascularization rates, in-stent thrombosis rates, target lesion failure, target vessel failure, and myocardial infarction.

**Conclusions:**

While bioresorbable stents are comparable to metallic stents in terms of cardiac mortality rates, they exhibit significant drawbacks that warrant clinical consideration.

## 1. Introduction

In patients with chronic stable coronary heart disease and significant extensive myocardial ischemia, interventional therapy is such as percutaneous coronary intervention (PCI) with stent placement plays a vital role and is considered a standard of care [1]. Early intervention is critical for patients at high risk of unstable angina and non-ST-elevation myocardial infarction, while immediate opening of the infarct-related vessel is essential in cases of acute ST-elevation myocardial infarction [2]. However, the use of traditional metallic vascular scaffolds, despite being widespread, can result in many adverse events, including accelerated atherosclerosis in the target vessel [3]. These shortcomings of metallic scaffolds continue to pose challenges to clinicians.

Bioresorbable vascular scaffolds (BVS) have emerged as a significant advancement in treating coronary lesions. Their main advantage lies in their ability to be completely absorbed by the body over a period of time, potentially reducing the incidence of late adverse events compared to metal stents [4]. Despite these advantages, recent clinical studies have reported an increase in adverse events with BVS, including ischemia-induced myocardial infarction and the need for early target lesion re-revascularization [4].

The aim of this study was to evaluate the published five-year follow-up outcome data of BVS versus conventional metallic stents. Our objective was to analyze the long-term efficacy and safety data, providing insights into their practical application and effectiveness in clinical settings. The findings aim to guide clinicians on the optimal use of BVS in treating coronary artery disease.

## 2. Methods

### 2.1 Search Strategy and Selection Criteria

This systematic review and meta-analysis was performed in accordance with the Preferred Reporting Items for Systematic Reviews and Meta-Analyses (PRISMA) Statement and was registered at the International Prospective Register of Systematic Reviews (number CRD42023445957).

We searched databases from the time of database construction to June 30th, 2023. The English databases included: PubMed, MEDLINE, EMBASE, Cochrane Library, WOS, Google Scholar, and CENTRAL; The Chinese databases included: CNKI, CBM, VIP, and Wanfang. There were no language restrictions. We used the following Medical Subject Headings (MeSH) terms and combined text: “Coronary Disease”, “Bioresorbable stent”, “Randomized controlled trials”. The complete search used for PubMed was: ([Coronary Disease{MeSH Terms} or {Coronary Diseases} or {Disease, Coronary} or {Diseases, Coronary} or {Coronary Heart Disease} or {Coronary Heart Diseases} or {Disease, Coronary Heart} or {Diseases, Coronary Heart} or {Heart Disease, Coronary} or {Heart Diseases, Coronary}] AND [Bioresorbable stent{MeSH Terms} or {Bioresorbable stents} or {Bioresorbable scaffold} or {Bioresorbable scaffolds} or {resorbable polymeric everolimus-eluting scaffold} or {Bioresorbable vascular scaffolds}] AND [Randomized controlled trials{MeSH Terms} OR {Randomized controlled trial} OR {RCTS} OR {RCT}]). We also considered all potentially eligible studies for review, irrespective of the primary outcome or language.

### 2.2 Study Selection and Data Extraction

We considered studies to be eligible for inclusion in this meta-study if they were randomized control trials performed in participants with coronary disease who required stenting with bioresorbable coronary stents or conventional coronary stents. We required that these trials reported 5-year clinical outcomes. Exclusion criteria included: non-randomized controlled trials, non-human trials, no clinically relevant outcomes, observation time of less than five years, reviews, conference abstracts, posters, letters, and case reports.

Two independent authors (YFF and HS) extracted the following data from each selected study: total number of participants, age, sex, trial duration, intervention strategies, outcomes, and other study characteristics. Discrepancies were resolved by consensus and discussion among the authors. If it could not be resolved through consensus and discussion, it was left to the third author (WBQ) to decide.

### 2.3 Assessment of Risk of Bias

Two independent reviewers (YFF and HS) assessed the quality of the included studies for risk of bias using the assessment tool described in the Cochrane Handbook for Systematic Reviews of Interventions [5]. All studies were assessed as low, unclear, or high risk of bias from the following six dimensions: (1) Random assignment Methods; (2) Allocation concealment; (3) Blinding of research subjects, implementation of treatment plans, and measurement of research results; (4) Incomplete outcome data; (5) No selective reporting of research results; (6) Other sources of bias.

### 2.4 Outcomes

The primary efficacy outcome of this study was rate of target lesion revascularization (TLR). The primary safety outcome was definite or probable rate of stent thrombosis (ST). Secondary outcomes included the rates of target lesion failure (TLF), target vessel failure (TVF), myocardial infarction (MI), and cardiac death. We defined TLR as any re-revascularization of the target lesion. The classification of definite or probable stent thrombosis followed the criteria established by the American Federation of Learned Research [6].

### 2.5 Statistical Analysis

RevMan5.4 software (Review Manager (RevMan) [Computer program]. Version 5.4. The Cochrane Collaboration, 2020. London, United Kingdom) was used in the meta-analysis, and odds ratio (OR) was used for dichotomous variables. In testing for heterogeneity among studies, if there was no heterogeneity (in Q test, *p* value > 0.10, $I^{2}$ < 50%), a fixed effect model was used; if there was heterogeneity (in Q test, *p* value ≤ 0.10, $I^{2}$ ≥ 50%) a random effects model was used.

### 2.6 Role of the Funding Source

This study was supported by the National TCM Inheritance and Innovation Program, NATCM’s Project of High-level Construction of Key TCM Disciplines (Yao Chun - Internal Medicine of TCM zyyzdxk-2023166). No commercial entity was involved. The funding source had no role in the study design, data collection, data analysis, data interpretation, or writing of the report. The corresponding author had full access to all the data in the study and had the final responsibility for the decision to submit the manuscript for publication.

## 3. Results

### 3.1 Study Selection and Characteristics

**Fig. 1.**
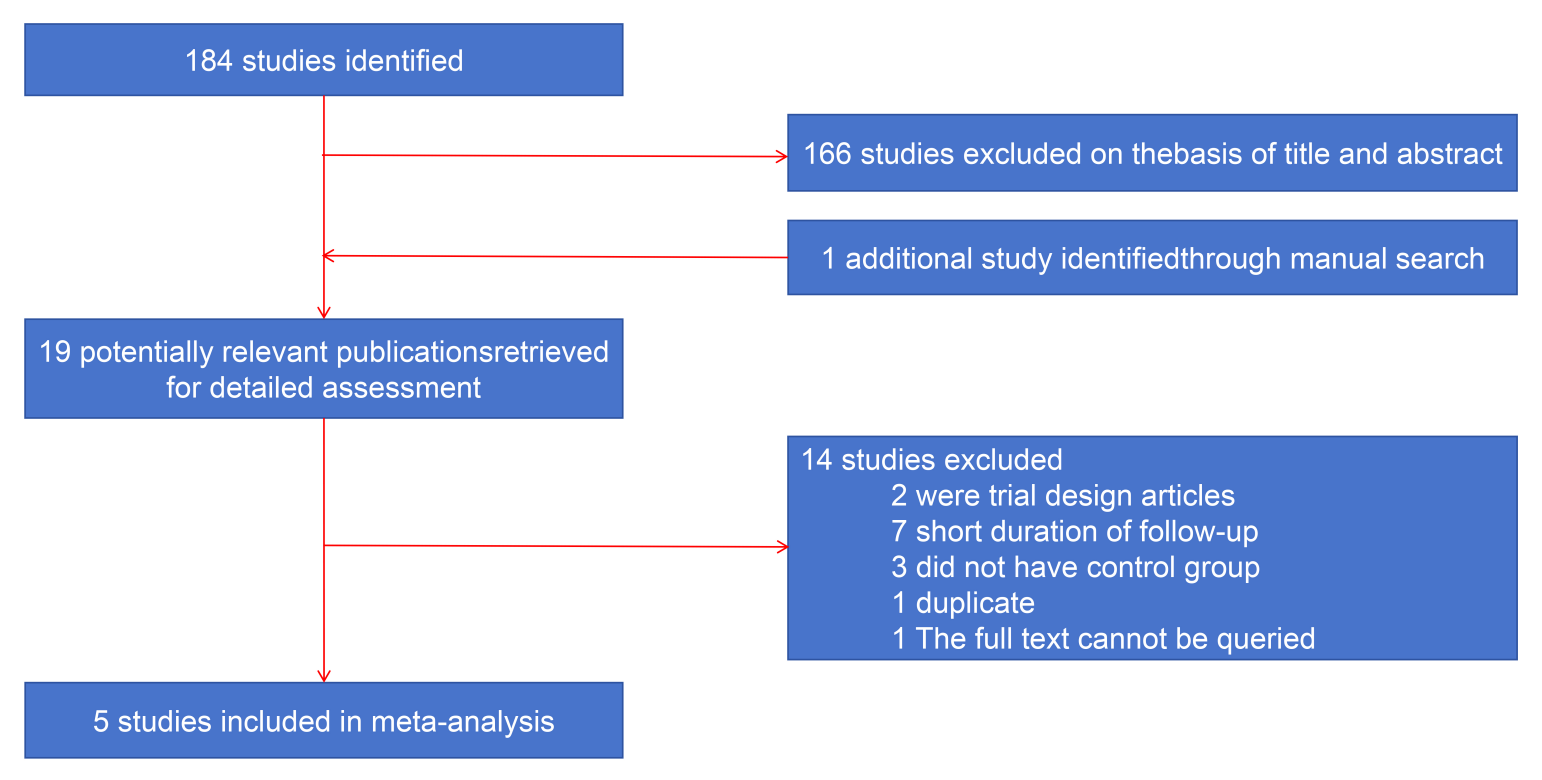
**Study selection process for the meta-analysis.** Initially, 184 studies were identified, from which 166 were excluded, followed by the addition of a subsequent study. After further evaluation, 19 potentially relevant publications were assessed in-depth. Of these, 14 studies were excluded for various reasons, leading to the inclusion of 5 high-quality randomized controlled trials in the final analysis.

During the study selection process we selected five high-quality papers from randomized controlled trials [7, 8, 9, 10, 11], published between 2019 and 2023 (Fig. 1). The characteristics of these five trials are shown in the Table 1 (Ref. [7, 8, 9, 10, 11]). The randomized controlled trials were from different countries and regions, with four being multicenter consortia [7, 8, 9, 11]. Together, these studies encompassed a total of 3886 participants in experimental groups and a total of 3209 in the control groups, with one study including over 1000 individuals in each group [11].

**Table 1.** **Basic characteristics of meta-analysis trials**.

Author	Publication time	Country	Sample size, n		Male gender, %		Age, y		Therapeutic measure		Outcomes
			Test group	Control group	Test group	Control group	Test group	Control group	Test group	Control group	
Dean [7]	2019	America	1322	686	/	/	/	/	BVS	EES	①②③④⑤⑥
Laura [8]	2022	Holland	924	921	/	/	/	/	Absorb BVS	XIENCE EES	①②③④⑤⑥
Ken [9]	2020	Japan	266	134	78.9	73.9	67.1 ± 9.4	67.3 ± 9.6	Absorb BVS	XIENCE CoCr-EES	①②③④⑤⑥
Gregg [11]	2023	America, Canada, Germany, Australia, Singapore	1296	1308	71.5	72.4	63.1 ± 10.1	62.2 ± 10.3	BVS	XIENCE CoCr-EES	①②③④⑤⑥
Sara [10]	2022	Holland	78	160	/	/	/	/	BVS	DES, BES, EES	①⑤⑥

Notes: ① Target lesion revascularization (TLR) rate. ② Stent thrombosis (ST) rate. ③ Target lesion failure (TLF) rate. ④ Target vessel failure (TVF) rate. ⑤ Myocardial infarction (MI) rate. ⑥ Cardiac death rate. BVS, bioresorbable vascular stent; EES, everolimus-eluting stent; DES, drug-eluting stent; BES, biolimus-eluting stent.

### 3.2 Results of Risk of Bias Assessment

**Fig. 2.**
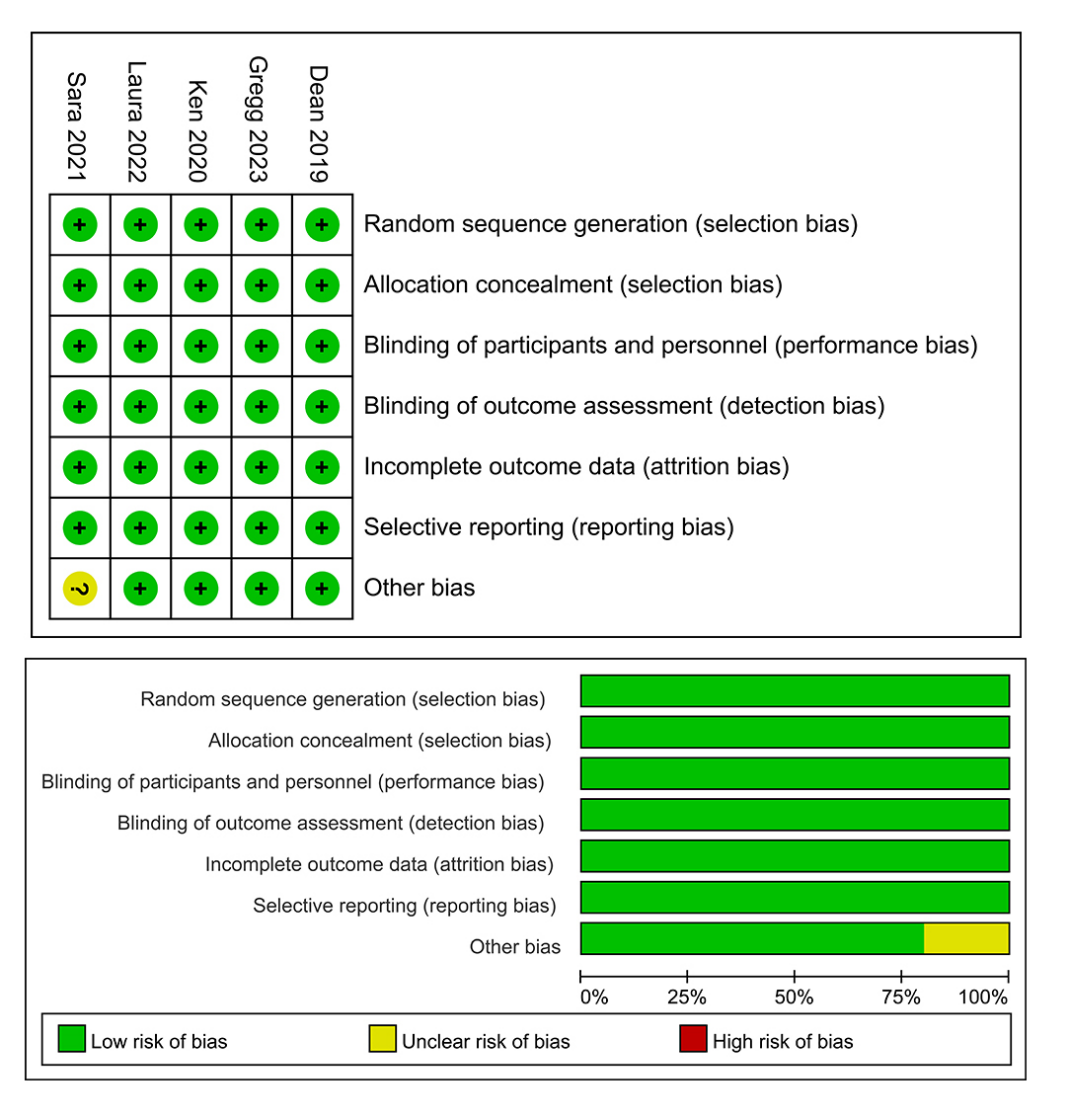
**Assessment of risk of bias for the selected studies. **This is a graphical presentation of the risk of bias as defined by Cochrane Handbook criteria. The graph visually represents the individual and overall assessments for each trial, highlighting studies with low risk and one study with an uncertain risk of bias due to inconclusive data on specific clinical parameters. This assessment ensures the reliability and validity of the study conclusions drawn from these trials.

We evaluated the quality of the selected studies based on the assessment tools described in the Cochrane Handbook for Systematic Reviews of Interventions [12]. Following a detailed reading of the full text and extensive data analysis, we assessed the studies based on objective and rational criteria. While All the trials were double blinded, had complete outcome data, and reported all pre-specified outcomes—indicating a low risk of bias in each of the evaluation metrics—only one study was categorized as having an uncertain risk of bias. This was due to its inability to provide definitive conclusions on certain clinical parameters. The evaluations of these biases are illustrated in Fig. 2.

### 3.3 Primary Efficacy Outcome

**Fig. 3.**
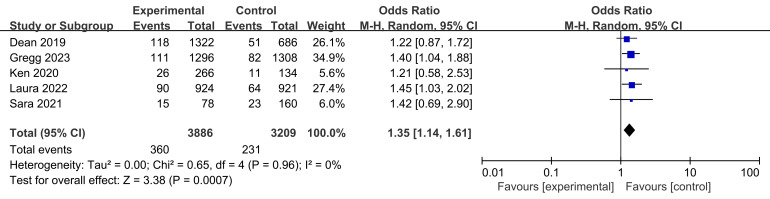
** Comparison of target lesion revascularization rates.** Fig. 3 compares the target lesion revascularization rates between BVS and metal stents across five trials. The graph includes data from 360 participants in the test group and 231 participants in the control group. Statistical analysis revealed no heterogeneity between groups, indicating consistent outcomes across studies. The combined odds ratio, demonstrates that BVS are associated with lower revascularization rates compared to metal stents. BVS, bioresorbable vascular stent; M-H, Mantel-Haenszel.

Target lesion revascularization rates were reported for all five trials, including a total of 360 participants in the experimental groups and 231 participants in the control groups (Fig. 3). The test for heterogeneity showed no significant differences between the experimental and control groups across the studies ($I^{2}$ = 0% and *p* = 0.96 > 0.05). However, the revascularization rate of target lesions treated with BVS was found to be lower than that of metal stents (*p* = 0.0007 < 0.05, Fig. 3).

### 3.4 Primary Safety Outcome

**Fig. 4.**
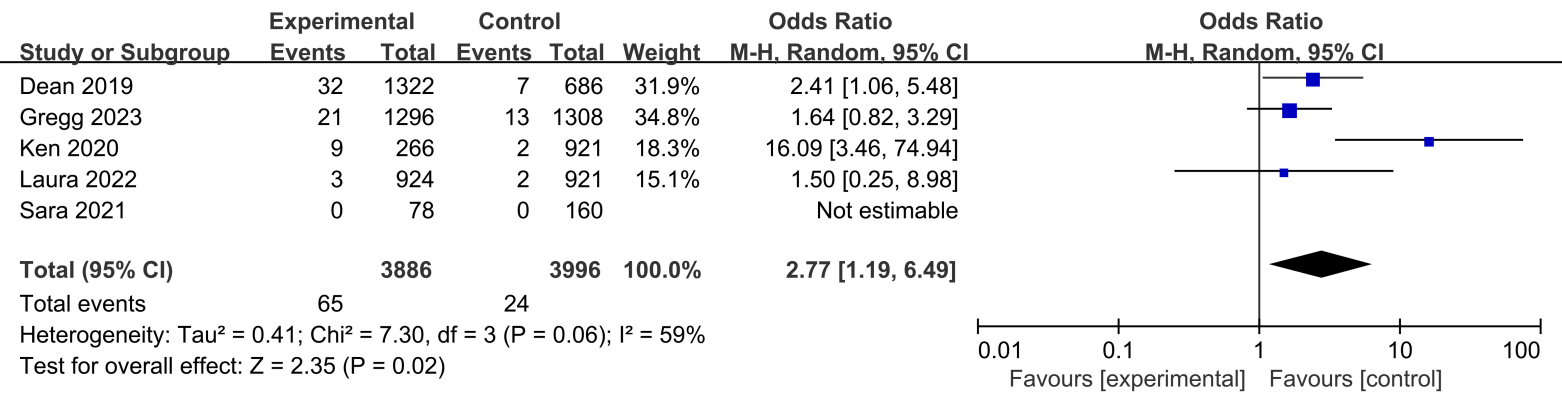
**Comparison of in-stent thrombosis rates.** Fig. 4 displays the in-stent thrombosis rates from four trials comparing BVS with metal stents. Data include outcomes from 65 patients in the test group and 24 patients in the control group. The heterogeneity test shows a moderate level of variability, indicating no significant differences in variability between groups. The combined odds ratio suggests a higher rate of in-stent thrombosis in patients treated with BVS compared to those receiving metal stents. BVS, bioresorbable vascular stent; M-H, Mantel-Haenszel.

The rate of in-stent thrombosis was reported in four studies, involving a total of 65 participants in the experimental group and 24 participants in the control group (Fig. 4). The heterogeneity test ($I^{2}$ = 59%, *p* = 0.06 > 0.05) suggested there was no heterogeneity in the results of the experimental and control groups across the studies. However, the rate of in-stent thrombosis was found to be higher after treatment with bioresorbable stents compared with metal stents (*p* = 0.02 < 0.05, Fig. 4).

### 3.5 Secondary Outcomes

**Fig. 5.**
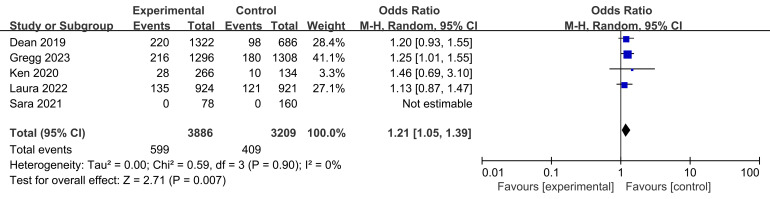
**Target lesion failure rates in CAD treatment.** Fig. 5 illustrates the target lesion failure rates from four studies comparing BVS with metallic scaffolds. It includes data from 599 patients in the test group and 409 in the control group. The heterogeneity test indicated no significant variability across the studies. Despite the homogeneity, the combined odds ratio demonstrates that BVS are associated with higher rates of target lesion failure when compared to metallic scaffolds in the treatment of CAD. BVS, bioresorbable vascular stent; CAD, coronary artery disease; M-H, Mantel-Haenszel.

Target lesion failure rates were reported in four studies, involving a total of 599 participants in the experimental groups and 409 participants in the control groups (Fig. 5). The test for heterogeneity showed no variability between the groups across the studies ($I^{2}$ = 0% and *p* = 0.90 > 0.05). However, the BVS treated participants had a higher rate of target lesion failure in the treatment of coronary artery disease (CAD) as compared with lesions treated with metallic scaffolds (*p* = 0.007 < 0.05, Fig. 5).

**Fig. 6.**
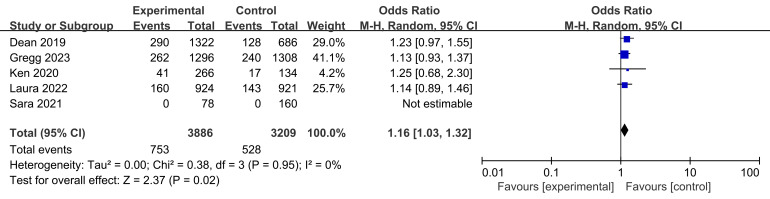
**Target vessel failure rates in CAD treatment.** Fig. 6 presents the target vessel failure rates from four studies comparing BVS to metallic scaffolds. This analysis included 753 patients in the test group and 528 in the control group. The heterogeneity test indicated no significant differences between the groups, demonstrating consistency across the studies. Despite this homogeneity, the significant combined odds ratio indicates a higher rate of target vessel failure in patients treated with BVS compared to those treated with metallic scaffolds in coronary artery disease management. BVS, bioresorbable vascular stent; CAD, coronary artery disease; M-H, Mantel-Haenszel.

Target vessel failure rates were reported in four studies, involving a total of 753 participants in the experimental groups and 528 participants in the control groups (Fig. 6). The test for heterogeneity revealed no differences between the groups across the studies ($I^{2}$ = 0% and *p* = 0.95 > 0.05). However, BVS treated participants had a higher rate of target-vessel failure in the treatment of CAD as compared with lesions treated with metallic scaffolds (*p* = 0.02 < 0.05, Fig. 6).

**Fig. 7.**
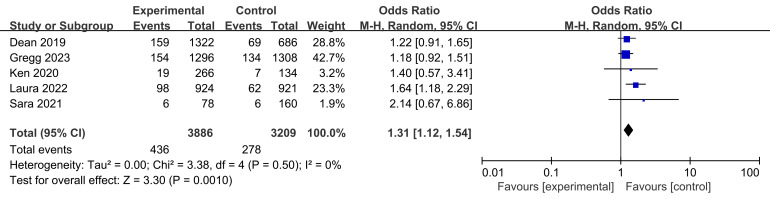
**Myocardial infarction recurrence rates following stent intervention.** Fig. 7 displays the myocardial infarction recurrence rates from five studies comparing BVS to conventional stents. The analysis included 436 patients in the test group and 278 in the control group. Although the heterogeneity test showed no variability among the studies, indicating consistent data, the significant combined odds ratio reveals a higher recurrence rate of myocardial infarction in patients treated with BVS compared to those receiving conventional stents. BVS, bioresorbable vascular stent; M-H, Mantel-Haenszel.

Myocardial infarction rates were reported in all five studies, involving a total of 436 participants in the experimental groups and 278 participants in the control groups (Fig. 7). The heterogeneity test found no variability between the groups across the studies ($I^{2}$ = 0%, *p* = 0.05). However, BVS treated participants had a higher myocardial infarction recurrence rate compared to conventional stents (*p* = 0.001 < 0.05, Fig. 7).

**Fig. 8.**
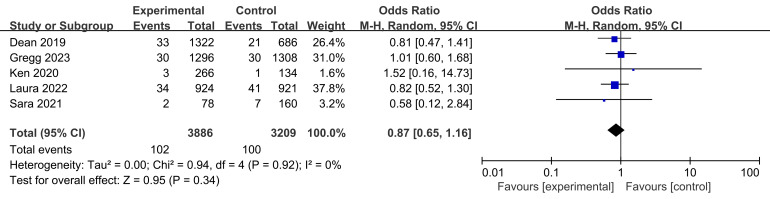
** Cardiac mortality rates at five years following stent treatment.** Fig. 8 compares cardiac mortality rates between bioresorbable and metallic stents over a five-year period in the treatment of coronary artery disease. The analysis included 102 patients in the test group and 100 in the control group. The heterogeneity test revealed no significant variability, confirming consistency across the studies. The post-combination odds ratio indicates no significant difference in cardiac mortality rates between the two stent types at the five-year mark. M-H, Mantel-Haenszel.

Cardiac mortality rates were reported for all five studies, involving a total of 102 participants in the experimental groups and 100 participants in the control groups (Fig. 8). The test for heterogeneity indicated no significant differences between the groups between the studies ($I^{2}$ = 0%, *p* = 0.92 >0.05). Additionally, we found no significant differences in cardiovascular mortality rates between the participants treated with bioabsorbable stents and those treated with metal stent compared with the metal stents over five years of clinical outcomes for CAD (*p* = 0.34 > 0.05, Fig. 8).

## 4. Discussion

As science and technology continue to advance in medicine, bioresorbable materials are gaining popularity for their biodegradability, plasticity, and ability to promote tissue regeneration [13]. Despite their widespread adoption and numerous benefits, clinical evaluations often reveal that these devices do not always meet expectations. For example, a clinical study comparing polylactate bioresorbable devices with traditional titanium bone and dental repairs found no significant difference between the two treatments in terms of bone and tooth stability [14]. Additionally, bioresorbable materials are utilized in various other medical applications, such as surgical sutures and extended-release drug capsules [15].

In recent years, BVS have become among the most widely used bioresorbable materials in medicine. This meta-analysis synthesized six outcome metrics from five-year observational data comparing BVS with metallic vascular scaffolds in high-quality randomized controlled trials for CAD interventions. To our knowledge, this is the first study to report a meta-analysis of the five-year clinical outcomes of BVS. The results indicate that bioabsorbable scaffolds were generally inferior to metallic scaffolds in five outcome metrics: target lesion reconstruction rates, in-stent thrombosis rates, target lesion failure rates, target vessel failure rates, and myocardial infarction rates. However, in the metric of cardiac mortality rates, bioabsorbable and metallic stents showed comparable results. Therefore, our study indicates that although bioabsorbable scaffolds are favored by many medical professionals and patients for their obvious advantage of complete absorption after a certain period of time [16], their disadvantages are also worthy of serious consideration and continued improvement.

Several factors contribute to these suboptimal results. The strut thickness significantly affects clinical outcomes, particularly in smaller blood vessels where thicker stents occupy a larger area of the target vessel. Clinical studies have revealed that the rate of stent thrombosis is significantly higher with bioresorbable stents in blood vessels with an internal diameter of less than 2.25 mm compared with that of conventional stents [17]. However, a clinical study demonstrated a very thin-strut bioresorbable stent implanted in a patient with CAD exhibited almost no thrombus formation from six months to three years following implantation, likely due to faster endothelialization and tissue resorption facilitated by the thinner strut [18]. Moreover, uneven stent degradation also poses a serious problem, as differential degradation rates may deform the stent and induce thrombosis [19]. In addition, the degradation process of bioresorbable stents reduces the local arterial pH, attracting inflammatory cells [20], and thereby increasing the likelihood of stent thrombosis.

Several studies have demonstrated that bioresorbable scaffolds often yield better outcomes one to two years after implantation when compared to metallic scaffolds [21], which has been attributed to improved healing following percutaneous coronary intervention [22, 23]. In contrast, numerous high-quality studies have reported poorer short-term clinical outcomes for bioresorbable scaffolds when compared to metallic scaffolds [24, 25, 26, 27, 28, 29], aligning with the results of our study. In general, the side effects associated with BVS are primarily related to changes in coagulation status after stent implantation. Therefore, focusing on antiplatelet therapy may be an effective method to improve the outcomes of individuals treated with these stents. Research indicates that the incidence of stent thrombosis is significantly lower following five years of dual antiplatelet therapy (DAPT) following BVS implantation compared to individuals not treated with DAPT [8]. In addition, the average patient age is another vital factor affecting the prognosis; older patients typically have poorer coagulation function, making the absence of a younger comparison group a notable shortcoming of the included studies.

Our study has several notable limitations. First, is sample size. Due to stringent inclusion and exclusion criteria, along with the necessity for five-year follow-up data, the resulting sample size of the study was relatively small. Second, is variability in intervention techniques. Differences in the techniques and technologies used across the included studies could contribute to variability in outcomes, especially if newer versions of bioresorbable scaffolds or advancements in metallic stent technology were introduced during the period of study. Third, lack of patient-level data. If the meta-analysis relied on aggregate data rather than individual patient data, this could limit the ability to perform more detailed subgroup analyses that might identify which patients benefit most from each type of stent. Fourth, confounding factors. The included studies might not have adequately controlled for all potential confounding factors, such as differences in medical management, patient comorbidities, or medication adherence, which could influence the outcomes. Fifth, reporting bias. There could be a reporting bias if studies with negative outcomes are less likely to be published, leading to an overestimation of the benefits of bioresorbable scaffolds. Finally, follow-up duration. While the five-year follow-up is comprehensive, longer-term outcomes beyond this period remain unknown, which could be crucial for understanding the durability and long-term safety of the interventions. We plan to perform further studies with a larger sample size to confirm the results from this meta-analysis.

## 5. Conclusions

The use of first-generation BVS for the treatment of coronary lesions is associated with inferior five-year clinical outcomes compared to traditional coronary stents, according to this meta-analysis of randomized controlled trials. Future studies should investigate whether newer bioresorbable stents, featuring thinner struts, can address these shortcomings.

### 5.1 Strengths and Limitations

The randomized controlled trials included in this study were all high-quality studies, ensuring robust data and reliable outcomes. However, a limitation is the small number of original randomized controlled trials included in this study, which could impact the breadth of the conclusions drawn.

### 5.2 What’s New

Our work is a systematic review and meta- analysis of the five-year outcomes of BVS for the treatment of CAD. To the best of our knowledge, comprehensive results from five-year studies have been limited, making our findings particularly valuable for advancing the understanding of long-term BVS performance.

## Data Availability

All data points generated or analyzed during this study are included in this article and there are no further underly-ing data necessary to reproduce the results.

## Supplementary Material

Supplementary material associated with this article can be found, in the online version, at https://doi.org/10.31083/j.rcm2507238.
